# A Comparative Study of Shock Absorption Capacities of Custom Fabricated Mouthguards Using a Triangulation Sensor

**DOI:** 10.3390/ma12213535

**Published:** 2019-10-28

**Authors:** Rūta Sarac, Julia Helbig, Juliane Dräger, Paul-Georg Jost-Brinkmann

**Affiliations:** Department of Orthodontics, Dentofacial Orthopedics and Pedodontics, Charité-Universitätsmedizin, Center for Dental and Craniofacial Sciences, Aßmannshauser Straße 4–6, D-14197 Berlin, Germany; ruta.sarac@gmail.com (R.S.); juliahelbig@gmx.de (J.H.); juliane.draeger@charite.de (J.D.)

**Keywords:** mouthguard, contact sports, dental trauma, triangulation laser sensor, shock absorption

## Abstract

This in-vitro study compares the shock absorption qualities of five mouthguard designs measured with a triangulation laser sensor during small hard object collisions. The aim was to investigate the impact of different labial designs on mouthguard performance. Methods: Five different custom-fabricated ethylene vinyl acetate (EVA) types of mouthguards with varying thickness and different labial inserts (polyethylene terephthalate glycol-modified (PETG), nylon mesh, air space) were tested with a triangulation laser sensor during different energy blows, generated with a pendulum testing device. The pendulum hits were applied to the center of a pivoted tooth crown in a custom-built upper jaw model. Measurements were executed with the mouthguards on the model and with no mouthguard as a negative control. Results: Tooth deflection was reduced with all mouthguards in comparison to no mouthguard. Increasing mouthguard thickness improved the mouthguards’ shock absorption capacities. Also, adding labial inserts increased their preventive qualities in ascending order: Mouthguard with a soft insert (nylon mesh), a hard insert (PETG), air space plus a hard insert (PETG). Conclusion: Increasing EVA foil thickness of a mouthguard, increasing labial thickness, and adding labial inserts (soft, stiff and air space) improve mouthguard shock absorption capabilities during small hard object collisions, thereby improving dental trauma prevention.

## 1. Introduction

Children, teenagers and adults face various sport-related orofacial injuries, which also can include teeth. It is important to prevent such types of injuries [1,2]. One option is a mouthguard. Mouthguards prevent injuries of oral hard and soft tissues and their complications [3,4]. Orofacial injuries can be a distressing event, which can cause physical as well as psychological problems to the victim, as they not only physically hurt but might also impair one’s looks [1]. Treatment might require long hours in a dentist’s chair, costing a lot of money, not to mention that the prophylactic and preventive check-ups might last for the rest of the patient’s life [5,6,7]. 

Investigations of mouthguard material qualities and the search for optimal mouthguard designs are fields which require further research. The aim of the study was to investigate the shock absorption capacities of different mouthguard designs and to compare their impact on a tooth in an in-vitro setup with the same energy blows. 

The objectives of this study are:

1. To manufacture five types of mouthguards with different designs from ethylene vinyl acetate (EVA), where two of them are single-layered and differ in thickness, the other three are laminated and have inserts in the frontal region (a hard insert, an air space and a nylon mesh insert);
2. to inflict different energy blows with a pendulum device on a pivoted tooth in an upper cast stainless steel model with and without a mouthguard;
3. to ascertain the dental deflections with a triangulation laser sensor and to assess the preventive qualities of the different mouthguard designs.

## 2. Materials and Methods 

### 2.1. Design of the Experiment

**Figure 1:**
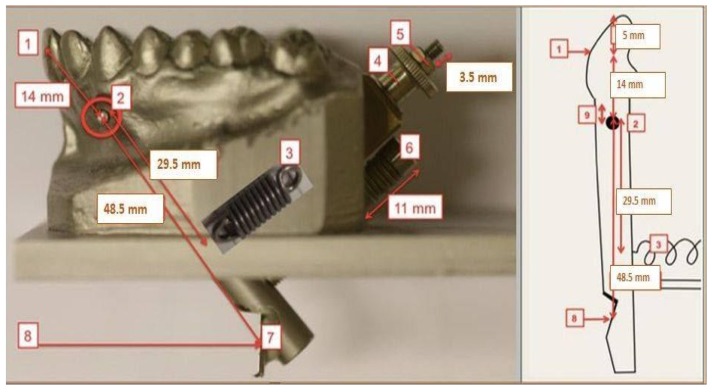
Stainless steel jaw model: (**1**) The pendulum strikes 14 mm above the rotation axis on the upper right incisor, (**2**) rotation axis, (**3**) tension spring (fixed to the extended tooth root inside the jaw model, 29.5 mm below the rotation axis), (**4**) screws, nuts and washers (holding the tension spring), (**5**) counter nut (fixes and prevents spring preload), (**6**) counter screw (prevents tooth movement to vestibular direction), (**7**) reflection foil, (**8**) laser beam hits 48.5 mm pointed to the reflection foil, (**9**) *limbus alveolaris* level 6.27 mm above the rotation axis and 2.73 mm below the gingival margin. Courtesy of Bochnig et al. [8].

This in-vitro study plan was designed according to Bochnig et al. [8]. A cast upper jaw model made from stainless steel with a pivoted upper right central incisor is attached to a base plate. The pivoted tooth is held in its normal position by a spring (Figure 1). The mouthguard to be examined is placed on the model and tested with a defined impact energy. The impact energy is induced to the mouthguard via a pendulum impactor mounted on the base plate, which was positioned such that the impact pendulum would hit the center of the pivoted tooth crown. The impact energy is varied by changing the deflection angle of the pendulum. The tooth movement caused by the impact is detected contactless with a triangulation laser sensor (optoNCDT 1750, Micro-Epsilon, Ortenburg, Germany). For this, the tooth’s extended root was fitted with a reflective surface (Reflective foil, Polytec GmbH, Berlin, Germany), which was mounted below the denture model and reflected the measuring beam of the laser. 

The cast stainless steel upper jaw model used in this study had been produced for the study of Bochnig et al. [8] based on an upper plaster model with well-aligned full complement of teeth after orthodontic therapy. The plaster model was duplicated in an iron-based alloy (1.4581 GX5CRNIMONB19-11-2, a corrosion-resistant high-grade steel) via injection molding. Upper central right incisor with an extended root was cast separately from the same alloy and pivotally mounted into the model with a horizontal rotation axis in the frontal plane (Figure 1). A tension spring with a spring constant of 11.297 N/mm (Z-100RI, Gutekunst + Co. KG, Metzingen, Germany) imitated the natural tooth mobility and positioned tooth 11 in its aligned position with no preload [8]. 

Spring force F is proportional to the deflection ∆l (Hooke’s law) and the proportionality factor D is called spring constant. From here, F = D ∆l equation applies [8]. “The required spring constant was calculated on the basis of an assumed tooth deflection of 0.01 mm initiated by a force of 0.5 N. With the given model geometry, it was calculated to be 11.25 N/mm” according to Bochnig et al. [8].

### 2.2. Mouthguard Production

**Figure 2:**
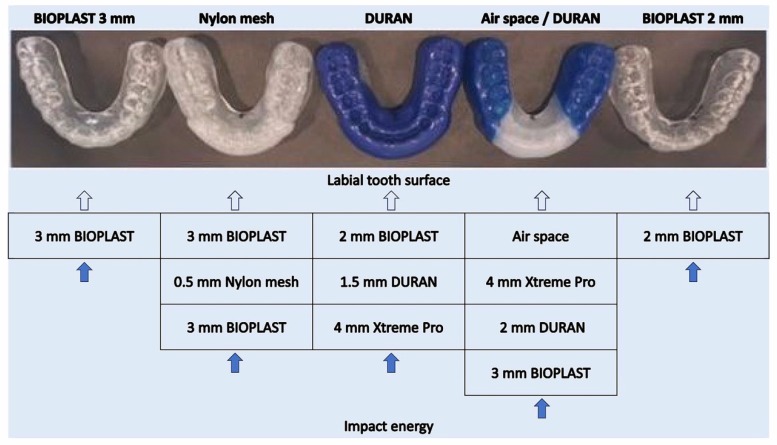
Schematic illustration of all five mouthguard designs with the direction of impact energy.

Five types of custom-made mouthguards were produced for shock absorption capacity comparison (two single-layer EVA mouthguards (BIOPLAST 2 mm and BIOPLAST 3 mm); three multi-layered EVA mouthguards) (Figure 2) [9,10]. Ten units of every type of mouthguard were manufactured. Materials used for every type of mouthguard used:

1. BIOPLAST 2 mm mouthguard (single-layer EVA mouthguard produced from Bioplast transparent 2 mm foil: Shore hardness A 85, Young’s modulus = 15 MPa, Item-No.: 3185, SCHEU-DENTAL GmbH, Iserlohn, Germany)
2. BIOPLAST 3 mm mouthguard (single-layer EVA mouthguard produced from Bioplast transparent 3 mm foil: Shore hardness A 85, Young’s modulus = 15 MPa, Item-No.: 3187, SCHEU-DENTAL GmbH, Iserlohn, Germany)
3. Air space/DURAN mouthguard (multi-layered EVA mouthguard produced from XTREME PRO 4 mm foil (BIOPLAST-XTREME PRO 4 mm: EVA, frontal segment (clear-transparent): Shore hardness A 92, tensile strength 20 MPa, Young’s modulus = 25 MPa; lateral segment (blue): Shore hardness A 85, tensile strength 18 MPa, Young’s modulus = 13 MPa, Item-No.: 3296, SCHEU-DENTAL GmbH, Iserlohn, Germany); and a layer of a BIOPLAST transparent 3 mm foil (EVA, Shore hardness A 85, Young’s modulus = 15 MPa, item-No.: 3354, SCHEU-DENTAL GmbH, Iserlohn, Germany) with an air space and a hard insert as a labial reinforcement (DURAN 2 mm foil (Polyethylenterephthalat-Glycol Copolyester (PET-G), Shore hardness D 78, Young’s modulus = 2200 MPa, Item-No.: 3436, SCHEU-DENTAL GmbH, Iserlohn, Germany)
4. NYLON MESH mouthguard (multi-layered EVA mouthguard produced from two BIOPLAST 3 mm (transparent) layers (BIOPLAST transparent 3 mm: EVA, Shore hardness A 85, Young’s modulus = 15 MPa, Item-No.: 3187, SCHEU-DENTAL GmbH, Iserlohn, Germany) and a Nylon mesh (0.5 mm) as a labial reinforcement (Nylon mesh 0.5 mm: Nylon mesh insert 0.5 mm (between the two layers of Bioplast, REF 3224.1, SCHEU-DENTAL GmbH, Iserlohn, Germany))
5. DURAN mouthguard (multi-layered EVA mouthguard produced from BIOPLAST 2 mm (blue) layer (BIOPLAST blue 2 mm: EVA Shore hardness A 85, Young’s modulus = 15 MPa, Item-No.: 3185, SCHEU-DENTAL GmbH, Iserlohn, Germany) and an XTREME PRO 4 mm (blue/transparent/blue) foil layer (BIOPLAST-XTREME PRO 4 mm: EVA, frontal segment (clear-transparent): Shore hardness A 92, tensile strength 20 MPa, Young’s modulus = 25 MPa; lateral segment (blue): Shore hardness A 85, tensile strength 18 MPa, Young’s modulus = 13 MPa, Item-No.: 3296, SCHEU-DENTAL GmbH, Iserlohn, Germany) with a hard labial reinforcement produced from DURAN 1.5 mm foil (DURAN 1.5 mm: Polyethylenterephthalat-Glycol Copolyester (PET-G), Shore hardness D 78, Young’s modulus = 2200 MPa, Item-No.: 3434, SCHEU-DENTAL GmbH, Iserlohn, Germany)

All details of exact mouthguard design and production are described in depth in the dissertation of Bochnig [10].

### 2.3. Pendulum Testing Device Construction, Triangulation Sensor Installment

**Figure 3:**
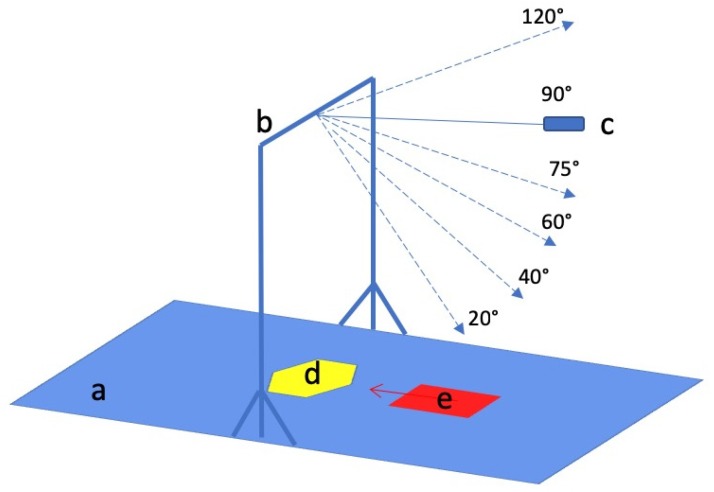
Schematic drawing of the pendulum testing device. (**a**) Pendulum testing device base-plate; (**b**) pendulum testing device frame; (**c**) pendulum; (**d**) stainless steel upper jaw model; (**e**) triangulation laser sensor. Dotted lines mark deflection angles used for different energy impact.

A pendulum testing device (Figure 3) was used to induce different impact energies to impinge the central area of the pivoted central incisor of the upper stainless steel model. The pendulum weighed 0.205 kg (constructed according to Bochnig et al. [8]). Different impact energies were produced with elongation angles of 20°, 40°, 60°, 75° and 90°, and with the mouthguard Air Space/Duran up to 120° (The pendulum elongation angles for the experiment were set according to the study by Bochnig et al. [8]). The impact energy rose with the elongation angle. A special frame was mounted to the pendulum testing device to induce the intended energy strike. To release the pendulum, a switchable release magnet was constructed.

A laser triangulation sensor was used to evaluate the impact of the different energy blows to the pivoted tooth. As the upper right incisor was hit by the pendulum, the laser sensor tracked the movement /displacement/ vibration of the elongated upper right central incisor root. An optoNCDT 1750 triangulation laser sensor from Micro-Epsilon (optoNCDT 1750, Micro-Epsilon, Ortenburg, Germany) was used during this experiment. It is designed for high-speed displacement, distance and position measurements. The triangulation laser sensor operates with a laser diode, which projects a visible light spot onto the measurement target. The reflected light is imaged by an optical receiving system onto a position-sensitive element. If the light spot changes its position, this change is imaged on the receiving element and evaluated. 

The construction was placed on a 15 mm thick antivibration rubber mat to avoid additional vibrations as much as possible.

### 2.4. Measurement Execution

Every mouthguard sample was tested with different pendulum energy blows from 20°, 40°, 60°, 75° and 90°; each mouthguard received 5 hits at every angle (between measurements, the mouthguard was removed and reset on the model again). Only Air Space/Duran mouthguards were tested with energy blows from 120° angle (also 5 hits per mouthguard). Each mouthguard type was manufactured 10 times in order to ensure representativity. The same experiments were also carried out as a negative control without a mouthguard (except for 120° angle), 10 hits from every angle. 

The formula for potential energy was used to calculate the kinetic energy of the pendulum at the point of impact: Ep = mgh where m is pendulum mass in kg, g—gravitational acceleration 9.81 ms^−2^, h—deflection of the pendulum [j] = rs [1 − cosj]. 

The digital signals from the laser sensor were transferred via RS422/USB converter (Micro-Epsilon, Ortenburg, Germany) to a laptop (Lenovo Thinkpad 20L50007GE, 8/256 SSD FHD LTE W10P W10P Germany) with MS Windows 10 software. The sensor and the converter were connected via the RS422 interface of the converter and data output was done via USB interface. When on, the sensor continuously measured the distance to the extended root of the pivoted tooth axis. 

Each measurement consisted of switching the measurement on the button of the laser, switching on the magnet switch to release the pendulum, recording the pendulum strike data on the measuring computer and switching off the magnet switch. 

### 2.5. Data Evaluation and Statistics

The influence of pendulum deflection (20°, 40°, 65°, 75°, 90° and 120°) was determined via Kruskal-Wallis + Mann-Whitney-U-Test by 2018 Microsoft Excel for Mac, Version 16.16.4 (Microsoft, Redmond, WA, USA) [10]. The maximum deflections of the impacted tooth were examined. 

Negative control measurements were carried out with ten repetitions of pendulum hits to the pivoted tooth without any mouthguard at 20°, 40°, 60°, 75° and 90° pendulum elongation angles. Ten pendulum strikes were carried out to the pivoted tooth crown from every pendulum elongation angle. The biggest pivoted tooth deflection during the impact was used for the representation of each specific energy blow impact on the tooth. 

Then the measurements with the mouthguards were carried out. From every single pendulum strike the biggest pivoted tooth deflection was taken for data analysis and comparison between mouthguards. The average of 5 repetitive pendulum strikes per every pendulum elongation angle was calculated as a representative of tooth deflection during a specific energy blow to the tooth while wearing a specific mouthguard sample. Every mouthguard design had 10 samples. Therefore, there were 10 average values of maximum tooth deflections for the shock absorption capability of each mouthguard design representation and comparison between designs and no mouthguard at all. 

**Figure 4:**
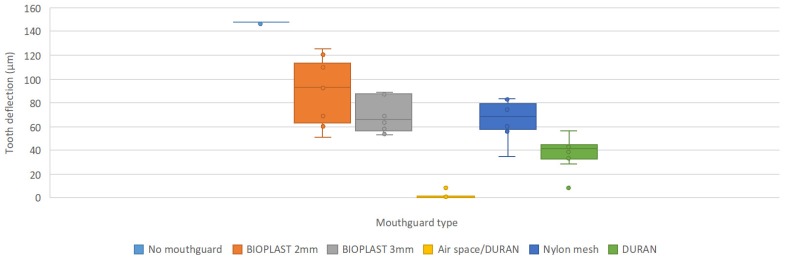
Box-whisker plots of all five mouthguard types’ average maximum deflection values and maximum deflection values with no mouthguard with 90° pendulum energy blows (generated energy: 1.15J).

Statistical tests and analysis (with a significance level of 0.05; confidence interval 0.95) were performed to test shock absorption capacities between different mouthguard designs. The results are presented as average values of maximum amplitudes of the deflection in µm of the prolongation of the impacted tooth (in Figure 4 this deflection is named tooth deflection).

All mouthguards significantly reduced tooth deflections at every angle compared to no mouthguard. 

## 3. Results

All replicas of mouthguards from BIOPLAST 2 mm and BIOPLAST 3 mm were deformed after energy blows from 75° pendulum elongation angles. However, they were continued to be tested under 90° energy blows.

The triangulation laser sensor measured the distance between the laser and the reflection foil (which was part of the pivoted tooth construction). The sensor recorded measurements every millisecond. The distance between the sensor and the pivoted tooth reflection foil at rest was 19.462 mm. The sensor recorded a minimal deflection/vibration of the tooth in the state of steadiness (no impact applied) which was measured to be 0.8 µm (noise, vibrations from other machines in the laboratory). 

Obtained results are represented in Table 1, Table 2, Table 3, Table 4, Table 5 and Table 6. The results of measurements are rounded to one digit (i.e., 4.730 µm ~ 4.7 µm). From Table 1, it is obvious that with increasing pendulum elongation angle, tooth deflection increases. 

Results of BIOPLAST 2 mm mouthguard tests (Table 2): The values of tooth deflection from 20 to 75° angles are increasing with growing pendulum elongation angle. However, tooth deflection values at 90° are smaller again.

With BIOPLAST 3 mm the tendency is similar like with BIOPLAST 2 mm (Table 3), where the deflection of the tooth is growing with the increasing impact energy (pendulum elongation angle) from 20°–75°. The 90° pendulum elongation angle measurements produced deflection values much smaller than the 75° elongation angle. 

Air space/DURAN mouthguard reveal very small pivoted tooth movements in comparison to tooth deflections with no mouthguard or BIOPLAST 2 mm or BIOPLAST 3 mm (Table 1, Table 2 and Table 3). There are, however, a few values which do not fit the tendency “the bigger the angle–the bigger the deflection”: The fourth mouthguard tooth deflection value in the 90° pendulum strike group drops to 0.7 µm from 1.1 µm from 75° angle. Also, the elongation for mouthguard 5 at 75° (0.7 µm) and 90° (8.0 µm) came as a surprise.

Table 4 also represents measurements additionally performed with 120° energy blows performed to Air Space/DURAN mouthguards. 

Nylon mesh tooth deflection values (Table 5) are inconsistent (see mouthguards 6 and 7). There are a few unexpected values if we compare mouthguard samples between themselves and obtained values between pendulum elongation angle: mouthguard 3, 60°: 5.4 µm, mouthguard 7, 60°: 1.0 µm, mouthguard 10: 40.6 µm. Whereas other mouthguard values from this angle range from 17.2–29.6 µm.

Mouthguard DURAN measurement results (Table 6) reveal an increasing tooth deflection with increasing pendulum elongation angle.

**Figure 5:**
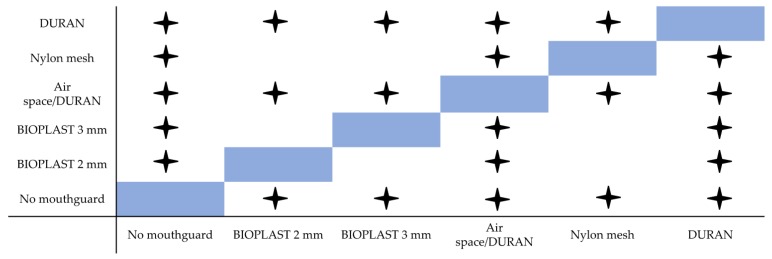
Statistical comparison of all five mouthguards and no mouthguard results with 90° pendulum energy blows (generated energy: 1.15 J).

Figure 4 illustrates the protective performance of the investigated mouthguards when hit the hardest (90°). Air space/DURAN does by far best followed by DURAN and the remaining 3 yielding similar results. These results can be displayed on a figure (Figure 5) comparing the statistical results of the box-whisker plots from Figure 4, where significant differences between different mouthguards or no mouthguard are marked with a star and no significant difference is not marked. 

## 4. Discussion

Leisure activities, as well as many sports, increase the risk of orofacial, in particular dental trauma [6,9,11,12,13,14,15]. In-vivo situations and stresses in the mouth differ in every case. Unfortunately, it is not possible to conduct an experimental in-vivo comparison of mouthguards to compare their effectiveness. In-vitro studies for mouthguard testing have also not yet been standardized [8,16]. Therefore, experimental setups and results vary substantially between studies. However, despite the non-homogenous results of numerous experimental studies, the protective benefit of individual mouthguards is undeniable [3,16,17,18,19,20]. 

The aim of the present in-vitro study was to test various individually produced mouthguards. Contradictory findings or unanswered questions in the literature regarding mouthguard thickness, protective effect of ethylene vinyl acetate (EVA) and hard liners or nylon mesh as reinforcement in the anterior region as well as air spaces were to be investigated. Different in-vitro studies have used a variety of measuring approaches: A finite element [21], an inertial measurement unit [22], a laser doppler vibrometer [8], and high-speed cameras [23]. The focus of Bochnig et al. [8] was hard, short impacts (0.07–2.85 J) on different individual mouthguards, to see which of the tested mouthguards deflection of the pivoted tooth test best. The main focus of the current study was the same in order to compare the results with Bochnig et al. [8] as the measurements were carried out with a different measuring device. Bochnig et al. [8] used a laser doppler vibrometer (Polytech OFV-301 Laser-Beam-Shutter and OFV-3000 Vibrometer Controller). In the present study, a triangulation laser sensor from Micro-Epsilon (optoNCDT 1750, Micro-Epsilon, Ortenburg, Germany) was used for tooth deflection measurements. There were a few changes made in the protocol of the experiment: Bochnig et al. used a pendulum with or without an additional weight to provide bigger impact energy (the pendulum with the added weight weighed 0.205 and 0.340 kg) resulting in impact energies between 0.07 and 2.85 J [8]. In the current study, the pendulum with an additional weight weighed 0.205 kg, so the produced impact energy was 0.07–1.72 J. It was decided that for data comparison achieved with two sensor technologies it is sufficient to repeat one series of experiments. 

As the most likely teeth to be injured during an orofacial trauma are maxillary central incisor [24], the study of Bochnig et al. [8] and the present investigation tested one upper central incisor. Only one central incisor was chosen due to the lack of space for the pivoted tooth construction in the stainless steel model [8]. 

The pendulum testing device was constructed according to Bochnig et al., therefore, the construction of the pendulum testing device had a wooden plate-base at the beginning [8]. However, due to disruptive vibrations which appeared during pilot measurements after the first mounting, the wooden base was exchanged for an aluminum plate. Preliminary tests carried out with this set-up showed that this experimental set-up was solid and appropriate for the planned measurements. 

Only custom-made mouthguards were tested in the present study because the materials and methods of Bochnig et al. [8] were followed and because custom-made mouthguards provide the best protection and comfort for the athletes [25].

The BIOPLAST and XTREME PRO foils used for mouthguard manufacture in the present study are EVA materials. Ethylene vinyl acetate (EVA) is the most frequently used mouthguard material [26,27] and has been studied in several experimental set-ups before [22,28,29,30,31,32]. The custom-made mouthguard impact energy absorption varies greatly due to multiple factors such as mouthguard design, thickness, and geometry of the EVA foil as well as the manufacturing process. Westerman et al. stated that the optimal thickness for EVA mouthguard material is around 4 mm, whereas further thickness increase reduces user comfort, increases speech restriction and interferes with respiratory efficiency [28]. Takeda et al. stated that a 3 mm + 3 mm EVA foil mouthguard reduces tooth deflection by 33%–48% [31].

The smaller the Young’s modulus of a mouthguard material is, the softer the pendulum impact will be: Relatively soft mouthguards (i.e., BIOPLAST (EVA) with Young’s modulus = 15 MPa) absorb impact forces, while hard mouthguard materials tend to disperse the forces [33]. However, if the material is too soft (EVA with Young’s modulus = 9 MPa), the mouthguard loses its protective effect. Very soft material provides hardly any resistance and is compressed by the impact object. This allows the impact object to destroy the mouthguard material and contact the tooth surface [33]. Very soft EVA was, therefore, not investigated.

The main focus of the study was to repeat the investigation by Bochnig [8] with a different measuring system and to estimate shock absorption capacities of different mouthguards and compare the impact outcome on the pivoted tooth with a mouthguard or no mouthguard. Despite deviations from physiological conditions, the stainless steel upper jaw model used in the study of Bochnig et al. and in the present study could provide comparisons of protective properties between various mouthguard types [8]. The results reveal that with increasing impact energy, the impact on the tooth (pivoted tooth deflection) is bigger (Table 1). The obtained results prove that individualized mouthguards reduce the impact of the shock applied to the tooth, because the deflection of the pivoted tooth with every replica of every mouthguard design was smaller than without a mouthguard with every energy blow tested. These results correspond well with the study of Bochnig et al., who also found that tooth deflection was significantly reduced by all mouthguards in comparison to measurements with no mouthguard [8]. However, big pivoted tooth deflection differences were recorded between the types of mouthguards in both studies: Bochnig et al. [8] and the present study. 

BIOPLAST 2 mm mouthguard revealed poor shock absorption capacities, actually the weakest of all five manufactured mouthguard designs. However, it has to be reminded that the deflections measured with 90° pendulum elongation energy blows, mouthguard performance was better than with the 75° angle. The reason for the changed tendency of “the bigger the angle = the bigger the deflection” might be explained by deformations which appeared in every BIOPLAST 2 mm mouthguard on 75° pendulum strikes: Indentations of the pendulum at the spot where the pendulum struck. The EVA material formed thicker rims around the indentation area, which may have improved the absorption of the later applied 90° energy blows. Also, Park et al. noted damage to single layer EVA mouthguards [34]. The study of Bochnig et al., however, did not mention any deformation of the mouthguards throughout the study [8]. All in all, our results confirm numerous previous studies stating the shock absorption capacity of single-layer mouthguards from 2 mm thick EVA foils to be very low [8,28,34,35,36]. 

From the results (Table 2 and Table 3) it is clear that mouthguards from thicker EVA foils are more effective in absorbing impact energy than thinner mouthguards from the same material, which is also confirmed by previously published studies [8,28,35]. Bochnig et al. [8] found BIOPLAST 3 mm to significantly reduce tooth deflection compared with BIOPLAST 2 mm mouthguards. 

In the current study, the Air space/DURAN mouthguard reduced tooth deflection much more than the other mouthguards. The combination of a 2 mm gap between the anterior teeth and the mouthguard with materials of different hardness (DURAN, XTREME PRO, BIOPLAST) and a high, labial layer thickness provided the best protection against the tested impact energies. Present study results and the results of Bochnig et al. [8] confirm earlier findings of Takeda et al. [31], who tested a similar type of mouthguard (1 mm distance between anterior teeth and mouthguard + 1 mm hard acrylic resin insert + 3 mm EVA + 3 mm EVA). Air space proves to be a very effective addition to individual mouthguard designs. 

When comparing the Nylon mesh mouthguards with BIOPLAST 2 mm mouthguards, Nylon mesh shock absorption capabilities at 20°, 40°, 60° and 75° pendulum elongation were more effective. However, the performance of Nylon mesh mouthguards was inconsistent (Table 5). The deflection ranges at 60°, 75° and 90° are very wide. Nylon mesh energy absorption performance seems to be similar to DURAN mouthguard at 20°, 40° and 60° energy level blows. Only from 75 and 90 degree angles, DURAN performance surpasses the Nylon mesh mouthguard. At 40°, 60° and 90°, the Nylon mesh results are similar to the results with BIOPLAST 3 mm (Table 2, Table 3, Table 4, Table 5 and Table 6). For now, from a clinical point of view, inconsistent test results do not ensure security. Instead, Air space/DURAN mouthguards should be considered despite their higher price. 

From the test results it can be seen that shock absorption capabilities decrease in the following order:


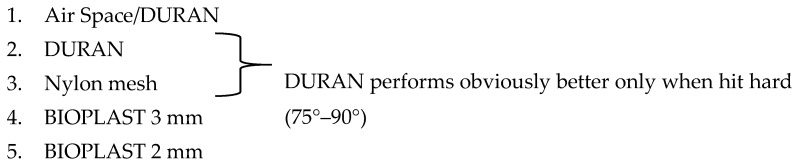


These present results correspond well with the findings of Bochnig et al. [8], despite the two very different measuring methods employed. Whenever there are so many measuring approaches available, it is just a hint that a single perfect measuring technique has not yet been developed. In other words, this study is not providing the absolute truth; we are just providing a comparison between mouthguards with one of these methods. It would be desirable to make further comparable studies with different measuring techniques.

There are certain limitations of the current study:

- It is an in-vitro study;
- Only one tooth was tested;
- No gingiva (intraorally there is flexibility of the surface of the gums).

The tested model is just one way to test the tooth under specific conditions with different mouthguards. It is not a perfect reflection of the intraoral situation.

The results reveal that by increasing the labial thickness of the mouthguard, the deflection of the impacted tooth is reduced. 

## 5. Conclusions

Several conclusions can be drawn within the limits of this in-vitro study:

1. All manufactured mouthguards had preventive qualities as tooth deflection was significantly decreased with all tested mouthguards in comparison to impacts without mouthguard.
2. BIOPLAST 2 mm and BIOPLAST 3 mm mouthguards revealed poor shock absorption capacities, their performance was not sufficient for dental trauma prevention: the deflection of the pivoted tooth was big even with low energy blows.
3. Increasing mouthguard labial thickness by approximately 1 mm (50%; BIOPLAST 2 mm vs BIOPLAST 3 mm) improves protection towards small hard object collisions with low impact energies (<1.72 J).
4. Combining different materials and using labial inserts improves mouthguard shock absorption capacities. Hard inserts for mouthguards (DURAN) serve better than soft inserts (Nylon mesh).
5. Air space in the front region of the mouthguard significantly improves mouthguard shock absorption capacities in comparison to any other tested protective mouthguard design. It improved shock absorption by up to 95% in comparison with the DURAN mouthguard at 90° pendulum hits.

## Figures and Tables

**Table 1:** NO MOUTHGUARD measurements: Maximum amplitude values of the pivoted tooth elongated root deflection obtained during measurements with energy blows from 20°–90°pendulum elongation angles (ten pendulum hits performed from every angle).

Pendulum Elongation Angle	Pivoted Tooth Deflection (µm) During the Pendulum Strikes									
20°	4.7	4.3	6.5	5.9	4.8	6.4	4.3	4.4	4.9	6.5
40°	53.9	53.9	45.6	52.3	50.6	55.8	44.1	49.7	45.9	54.6
60°	85.2	84.3	81.6	65.8	68.8	69.8	74.6	73.0	78.6	83.5
75°	145.2	142.6	146.7	144.3	146.0	144.1	142.1	143.7	138.9	141.7
90°	147.9	146.9	147.9	147.9	147.9	147.9	147.9	147.9	147.9	148.0

**Table 2:** BIOPLAST 2 mm mouthguard measurements: Average values of maximum amplitudes (mean of 5 measurements per mouthguard and angle) of the pivoted tooth (reflection foil) deflection obtained during measurements with energy blows from 20°–90° pendulum elongation angles.

Pendulum Elongation Angle	Pivoted Tooth Deflection (µm) During the Pendulum Strikes of 10 Mouthguard Samples (n = 10)									
20°	2.0	1.8	2.0	1.5	1.9	1.6	1.6	1.9	1.7	2.1
40°	12.8	20.2	14.8	12.4	14.9	10.1	11.1	8.9	8.3	12.4
60°	40.9	30.5	22.7	31.7	33.3	23.8	35.3	38.1	40.7	37.6
75°	123.4	127.2	124.2	127.5	127.0	124.8	123.5	126.0	128.0	127.5
90°	68.6	60.5	63.6	52.2	51.0	92.4	93.9	125.2	120.9	109.6

**Table 3:** BIOPLAST 3 mm mouthguard measurements: Average values of maximum amplitudes (mean of 5 measurements per mouthguard and angle) of the pivoted tooth (reflection foil) deflection obtained during measurements with energy blows from 20°–90° pendulum elongation angles.

Pendulum Elongation Angle	Pivoted Tooth Deflection (µm) During Pendulum Strikes of 10 Mouthguard Samples (n = 10)									
20°	1.2	1.7	1.8	1.6	1.8	1.5	1.8	1.1	1.9	1.6
40°	2.7	2.1	2.3	2.4	2.1	2.1	2.1	2.1	2.3	2.1
60°	31.4	11.4	12.5	10.6	11.6	10.3	12.1	11.8	9.0	11.9
75°	116.3	103.2	114.5	115.6	114.4	119.1	114.1	117.7	117.3	116.8
90°	57.7	53.7	60.3	63.1	53.2	68.6	87.8	89.0	71.5	86.9

**Table 4:** Air space/DURAN mouthguard measurements: Average values of maximum amplitudes (mean of 5 measurements per mouthguard and angle) of the pivoted tooth (reflection foil) deflection obtained during measurements with energy blows from 20°–120° pendulum elongation angles.

Pendulum Elongation Angle	Pivoted Tooth Deflection (µm) During Pendulum Strikes of 10 Mouthguard Samples (n = 10)									
20°	0.2	0.2	0.2	0.2	0.2	0.2	0.2	0.2	0.2	0.2
40°	0.4	0.3	0.4	0.3	0.3	0.4	0.3	0.3	0.3	0.3
60°	0.5	0.5	0.5	0.5	0.5	0.5	0.5	0.4	0.4	0.4
75°	0.6	0.6	1.1	0.8	0.7	0.7	0.6	0.5	0.4	0.5
90°	0.9	0.8	0.7	0.9	0.8	8.0	0.7	1.2	0.5	0.7
120°	6.2	2.1	1.8	1.9	2.2	2.0	1.1	2.5	0.7	0.8

**Table 5:** Nylon mesh mouthguard measurements: Average values of maximum amplitudes (mean of 5 measurements per mouthguard and angle) of the pivoted tooth (reflection foil) deflection obtained during measurements with energy blows from 20–90 degree pendulum elongation angles.

Pendulum Elongation Angle	Pivoted Tooth Deflection (µm) During Pendulum Strikes of 10 Mouthguard Samples (n = 10)									
20°	0.9	1.2	0.8	0.5	1.1	1.3	1.1	0.4	1.1	0.3
40°	1.6	1.7	1.0	0.4	1.3	0.9	1.0	0.5	1.1	0.5
60°	28.9	29.6	5.4	17.2	22.8	21.5	23.2	1.0	24.9	4.3
75°	44.8	69.7	52.7	56.1	60.1	63.8	50.1	11.5	69.5	40.6
90°	58.3	82.6	55.8	74.8	63.0	74.3	77.3	36.1	83.0	60.0

**Table 6:** DURAN mouthguard measurements: Average values of maximum amplitudes (mean of 5 measurements per mouthguard and angle) of the pivoted tooth (reflection foil) deflection obtained during measurements with energy blows from 20°–90° pendulum elongation angles.

Pendulum Elongation Angle	Pivoted Tooth Deflection (µm) During Pendulum Strikes of 10 Mouthguard Samples (n = 10)									
20°	0.6	1.0	0.5	0.5	0.9	0.7	0.6	0.5	0.5	1.0
40°	1.0	0.6	0.7	1.1	0.9	2.7	1.2	1.1	1.1	0.9
60°	4.9	12.8	0.9	13.1	5.3	22.2	9.6	8.4	7.7	5.9
75°	19.5	31.1	2.2	36.9	33.3	50.1	36.8	37.4	32.6	35.2
90°	33.3	42.6	8.0	45.0	42.6	56.1	43.5	28.2	40.4	38.4
